# SARS-CoV-2-Induced Immunosuppression: A Molecular Mimicry Syndrome

**DOI:** 10.1055/s-0042-1748170

**Published:** 2022-07-14

**Authors:** Darja Kanduc

**Affiliations:** 1 Department of Biosciences, Biotechnologies and Biopharmaceutics, University of Bari, Bari, Italy

**Keywords:** SARS-CoV-2, immunosuppression, molecular mimicry, cross-reactivity, NFKB, V(D)J RAG proteins

## Abstract

**Background** Contrary to immunological expectations, decay of adaptive responses against severe acute respiratory syndrome-coronavirus-2 (SARS-CoV-2) characterizes recovered patients compared with patients who had a severe disease course or died following SARS-CoV-2 infection. This raises the question of the causes of the virus-induced immune immunosuppression. Searching for molecular link(s) between SARS-CoV-2 immunization and the decay of the adaptive immune responses, SARS-CoV-2 proteome was analyzed for molecular mimicry with human proteins related to immunodeficiency. The aim was to verify the possibility of cross-reactions capable of destroying the adaptive immune response triggered by SARS-CoV-2.

**Materials and Methods** Human immunodeficiency–related proteins were collected from UniProt database and analyzed for sharing of minimal immune determinants with the SARS-CoV-2 proteome.

**Results** Molecular mimicry and consequent potential cross-reactivity exist between SARS-CoV-2 proteome and human immunoregulatory proteins such as nuclear factor kappa B (NFKB), and variable diversity joining V(D)J recombination-activating gene (RAG).

**Conclusion** The data (1) support molecular mimicry and the associated potential cross-reactivity as a mechanism that can underlie self-reactivity against proteins involved in B- and T-cells activation/development, and (2) suggest that the extent of the immunosuppression is dictated by the extent of the immune responses themselves. The higher the titer of the immune responses triggered by SARS-CoV-2 immunization, the more severe can be the cross-reactions against the human immunodeficiency–related proteins, the more severe the immunosuppression. Hence, SARS-CoV-2-induced immunosuppression can be defined as a molecular mimicry syndrome. Clinically, the data imply that booster doses of SARS-CoV-2 vaccines may have opposite results to those expected.

## Introduction

Notwithstanding the massive anti-SARS-CoV-2 vaccination campaign, breakthrough infections that can progress to severe illness have occurred in repeatedly vaccinated people. 1 Possibly, such an undesired effect might result from SARS-CoV-2-induced immunosuppression as suggested by numerous clinical data. Indeed, as examples among the many as follows:

- Analyses of blood samples from fully vaccinated health care workers showed that antibody (Ab) titers increased significantly at 5 weeks after first vaccination but decreased rapidly within 4 months after second vaccination. 2
- Individuals who received two doses of vaccine had a gradual increase of higher risk of SARS-CoV-2 infection with time elapsed since the second vaccine dose. 3
- Individuals who received the vaccine had different kinetics of Ab levels compared with patients who had been infected with the SARS-CoV-2, with higher initial levels but a much faster exponential decrease in the first group. 4
- Following two vaccine doses, SARS-CoV-2 antispike immunogammaglobulin (IgG) levels waned with an estimated half-life of 45 days and a decrease below detection level within 225 days. 5
- Ab decay following natural infection has been reported 6 7 8 with antinucleocapsid Abs declining more rapidly than antispike Abs. 9 10
- As reviewed by Lee and Oh, 11 a rapid decline in anti-SARS-CoV-2 Ab levels was found in novel coronavirus disease 2019 (COVID-19) patients with mild symptoms or asymptomatic individuals, 7 12 while higher Ab titers associated with severe COVID-19 manifestations. 13 14 15 16 In particular, IgG Abs against SARS-CoV-2 nucleocapsid were found to be significantly lower in mild SARS-CoV-2 infected patients 9 and declined more rapidly than spike Abs, 6 9 10 17 with antinucleocapsid IgG seropositivity higher in pneumonia patients than in nonpneumonia/asymptomatic patients. 18
- High concentration of IgG against the nucleocapsid protein characterized poor outcome in COVID-19 and caused a three-fold increase in risk of admission to the medical intensive care unit. 19
- Suboptimal SARS-CoV-2 − specific CD8+ T-cell response has been reported, 20 and suppressed CD8 ^+^ T-cell differentiation was found to be associated with prolonged SARS-CoV-2 positivity. 21
- Moreover, SARS-CoV-2 infection of children leads to a mild illness with significantly lower CD4+ and CD8+ T-cell responses to SARS-CoV-2 structural and ORF1ab proteins compared with infected adults. 22
- Lower than expected T-cell responses have been reported in healthy double vaccinated individuals. 23

However, in spite of the multitude of such prominent and various clinical data, notwithstanding viral-induced immunosuppression is a phenomenon known and discussed for decades and historically dating back to observations by von Pirquet in 1908, 24 and further references therein, it is disappointing to admit our scanty knowledge of the molecular basis and mechanism that lead to immune decay following viral infections. In the case under study, the cardinal question that remains unanswered and till now, to the best of the author's knowledge, has not been clearly posed is the following. Why the anti-SARS-CoV-2 humoral and cellular immune responses decline in recovered, asymptomatic, and mild SARS-CoV-2 patients while remain higher in severe patients? Actually, the immune responses triggered by SARS-CoV-2 should be high titer and long lasting in recovered patients in that the immune responses are supposed to ensure the eradication of the pathogen and to prevent/resolve diseases associated with the infection. And, vice versa, the immune responses should be low titer and waning in patients with severe or fatal COVID-19 course. Today, this question is relevant also in light of the fact that repeated booster doses of SARS-CoV-2 vaccines are being proposed for evaluation to enhance the immune response of the human host. 5

In this clinical context and on the basis of reports 25 26 that documented a high level of molecular mimicry between SARS-CoV-2 and human proteins, the hypothesis was tested here according to which the anti-pathogen immune responses are not exclusively directed against the virus but actually can cross-react with human proteins, in this way unleashing a self-attack against the human host and causing immunosuppression and the associated pathologic consequences, that is, uncontrolled infections, increased risk of cancer, and cardiovascular diseases, inter alia. 24

Precisely, taking into consideration data obtained using as a research model the Measles virus–induced immunosupression, 27 the hypothesis has been tested that immune responses against SARS-CoV-2 have the potential to cross-react with human proteins that—when altered, mutated, deficient, deleted, or otherwise functioning improperly—lead to immunosuppression.

To prove/disprove the cross-reactivity paradigm, the present study comparatively analyzed the entire SARS-CoV-2 proteome and human proteins involved in immunodeficiencies searching for common amino acid (aa) sequences. Using the pentapeptide as the basic measurement unit of antigenicity and immunogenicity, 28 29 30 31 32 33 34 35 36 37 38 39 40 41 42 43 44 45 46 47 sequence analyses revealed peptide commonalities that are susceptible of generating cross-reactions, thus feasibly explaining the immunosuppression associated with SARS-CoV-2 passive/active infection and its increase following repeated anti-SARS-CoV-2 vaccinations.

## Materials and Methods

The analyzed 10 SARS-CoV-2 proteins were derived from Wuhan-Hu-1, GenBank: MN908947.3, and are listed with the National Center for Biotechnology Information (NCBI) ID protein in parentheses as follows: ORF1ab polyprotein (QHD43415.1), spike glycoprotein (QHD43416.1), ORF3a protein (QHD43417.1), envelope protein (QHD43418.1), membrane glycoprotein (QHD43419.1), ORF6 protein (QHD43420.1), ORF7a protein (QHD43421.1), ORF8 protein (QHD43422.1), nucleocapsid phosphoprotein (QHD43423.2), and ORF10 protein (QHI42199.1).

Human immunodeficiency–related proteins were randomly collected from the UniProt database ( *www.uniprot.org/* ) 48 49 using “immunodeficiency hypogammaglobulinemia AND reviewed” as keywords. Thirty-eight human proteins were obtained and are listed in Supplementary Table S1.

Methodologically, the primary sequence of the SARS-CoV-2 proteins was dissected into pentapeptides offset by one residue (i.e., MESLV, ESLVP, SLVPG, LVPGF, and others) and the resulting viral pentapeptides were analyzed to find perfect matches within the 38 human proteins which, when altered, relate to immunodeficiencies. Protein information resource peptide match ( *research.bioinformatics.udel.edu/peptidematch/index.jsp* ) and peptide search ( *www.uniprot.org/peptidesearch/* ) programs that are available at UniProt ( *www.uniprot.org/* ) were used. 48 49 CoV controls are as follows, with NCBI:txid in parentheses: Middle East Respiratory Syndrome (MERS)-CoV (1335626), Human (H) CoV-229E (11137), and HCoV-NL63 (277944).

The human proteins involved in the peptide sharing (i.e., 32) were analyzed for functions/diseases using UniProt, PubMed, and OMIM ( *www.omim.org/* ) public resources. Human proteins are given by UniProt entry and/or UniProt name.

The immunological potential of the peptide sharing was analyzed by searching the Immune Epitope DataBase (IEDB; *www.iedb.org/* ) 50 for SARS-CoV-2-derived immunoreactive epitopes hosting the shared pentapeptides. Only unmodified epitopes ≤15 mers were considered. Given the size of the available data (i.e., 9,917 SARS-CoV-2-derived epitopes as of January 2022), analyses were limited to the peptide sharing involving nuclear factor kappa B1 (NFKB1) and NFKB2.

## Results and Discussion

Pentapeptide sharing between SARS-CoV-2 proteins and human immunodeficiency–related proteins was analyzed using pentapeptide as a sequence probe because a peptide grouping formed by five aa residues defines a minimal immune determinant underlying the specific interaction of an antigen with B-cell receptor (BCR) and T-cell receptor (TCR). 28 29 30 31 32 33 34 35 36 37 38 39 40 41 42 43 44 45 46 47 The results are displayed in Table 1 .

**Table 1:** Peptide sharing between the SARS-CoV-2 proteome and human immunodeficiency–related proteins

Viral protein a	Human immunodeficiency-related protein b	Shared peptides
ORF1ab	ALG12: Dol-P-Man:Man(7)GlcNAc(2)-PP-Dol α-1,6-mannosyltransferase	LCLFL, VVNAA
	BCL10: B-cell lymphoma/leukemia 10	ATNNL
	BTK: tyrosine-protein kinase BTK	DEFIE, EIDPK
	C2TA: MHC class-II transactivator	LPSLA, VLLIL, AELAK, EVLLA
	CAR11: caspase recruitment domain-containing protein 11	LGSLA, TTLNG, GSLPI, RKQIR, LQPEE, LDDDS
	CD19: B-lymphocyte antigen CD19	PKGPK, ETGLL
	CD20: B-lymphocyte antigen CD20	PSTQY
	CD27: CD27 antigen	GVSFS
	CR2: complement receptor type 2	LQGPP, GFTLK, FTLKG
	CTLA4: cytotoxic T-lymphocyte protein 4	GTSSG
	CXCR4: C-X-C chemokine receptor type 4	LLLTI
	I2BP2: interferon regulatory factor 2-binding protein 2	PTLVP, AKPPP
	IKZF1: DNA-binding protein Ikaros	SDRVV, ESLRP, VSTSG, GLPGT, ENLLL
	IRF9: interferon regulatory factor 9	EDQDA, DTTEA
	KPCD: protein kinase C delta type	GSSKC, NLIDS, LVKQG, LDNVL, CDHCG
	LAT: linker for activation of T-cells family member 1	QFKRP
	MOES: Moesin	SEAVE
	NFKB1: nuclear factor NF-κ-B p105 subunit	DLSVV, KAALL, ALRQM, KTPKY, TPKYK, ISLAG
	NFKB2: nuclear factor NF-κ-B p100 subunit	PKDMT, NNLGV, SVGPK, ANVNA, DFKLN
	NS1BP: influenza virus NS1A-binding protein	GIATV, ATVQS, SAAKK, EMLAH, IIGGA, EEEEF
	P85A: phosphatidylinositol 3-kinase regulatory subunit α	KPRPP, LKHFF, SLKEL, IQLLK, LRKGG
	RAG1: V(D)J recombination-activating protein 1	VSAKP, KTPEE, ILSPL
	RAG2: V(D)J recombination-activating protein 2	NSQTS, VSSAI, KQVVS, FDTYN, NIALI
	RFX5: DNA-binding protein RFX5	PLKSA, EVPVS
	RFXK: DNA-binding protein RFXANK	FTPLI, SVSSP
	TR13C: tumor necrosis factor receptor superfamily 13C	PAPRT, RDAPA, AGEAA
	TRNT1: CCA tRNA nucleotidyltransferase 1, mitochondrial	LQQLR
	VAS1: V-type proton ATPase subunit S1	SDRDL, GSVAY, VAYFN, LKSED
	XIAP: E3 ubiquitin-protein ligase XIAP	SQTSL, HAAVD, LARAG
Spike	ALG12: Dol-P-Man:Man(7)GlcNAc(2)-PP-Dol α-1,6-mannosyltransferase	TQLPP, PRTFL
	CAR11: caspase recruitment domain-containing protein 11	TNSFT, SNNLD
	CR2: complement receptor type 2	TFKCY, SYECD
	I2BP2: interferon regulatory factor 2-binding protein 2	TLLAL, LLALH
	NFKB1: nuclear factor NF-κ-B p105 subunit	LVRDL
	NFKB2: nuclear factor NF-κ-B p100 subunit	ALLAG
	TR13B: tumor necrosis factor receptor superfamily 13B	VPAQE
ORF3a	C2TA: MHC class-II transactivator	GEIKD
	CAR11: caspase recruitment domain-containing protein 11	ITSGD
	CD27: CD27 antigen	TIPIQ
	IKZF1: DNA-binding protein Ikaros	NLLLL
	NFKB1: nuclear factor NF-κ-B p105 subunit	LLLVA, LLVAA, LVAAG
Envelope	CD70: CD70 antigen	VTLAI
	SP110: Sp110 nuclear body protein	LLVTL
	VAS1: V-type proton ATPase subunit S1	VLLFL
Membrane	CAR11: caspase recruitment domain-containing protein 11	HSSSS
	TRNT1: CCA tRNA nucleotidyltransferase 1, mitochondrial	LRIAG
	VAS1: V-type proton ATPase subunit S1	KLGAS
ORF7	** –**	** –**
ORF8	** –**	** –**
Nucleocapsid	C2TA: MHC class-II transactivator	FAPSA
	CD19: B-lymphocyte antigen CD19	GPQNQ
	CTLA4: cytotoxic T-lymphocyte protein 4	PPTEP
	NFKB1: nuclear factor NF-κ-B p105 subunit	DSTGS, LLDRL, ELIRQ
	NFKB2: nuclear factor NF-κ-B p100 subunit	RPQGL
	RFX5: DNA-binding protein RFX5	RNSTP
	SP110: Sp110 nuclear body protein	GTWLT
ORF10	** –**	** –**

Abbreviation: SARS-CoV-2, severe acute respiratory syndrome-coronavirus-2.

a Viral proteins described under methods.

b Human proteins given by UniProt entry and name. Disease association and references are available at UniProt, PubMed, and OMIM public databases.

Next, to evaluate the specificity of the peptide commonalities described in Table 1 , the shared pentapeptides (that is, 118) were analyzed for occurrences in the control CoV proteomes MERS-CoV, hCoV-229E, and hCoV-NL63 ( Table 2 ).

**Table 2:** Quantitation of the pentapeptide sharing between CoV proteomes and human immunodeficiency − linked proteins

CoV	Number of shared pentapeptides
SARS-CoV-2	118
MERS-CoV	−
HCoV-229E	2
HCoV-NL63	3

Abbreviation: HCoV, human coronavirus; MERS-CoV; Middle East respiratory syndrome-coronavirus; SARS-CoV-2, severe acute respiratory syndrome-coronavirus-2.

In summary, Tables 1 and 2 show that following points:

- One hundred and eighteen pentapeptides are shared between the SARS-CoV-2 proteins and the human immunodeficiency–related proteins analyzed in this study. Mathematically, such a high degree of peptide commonality is unexpected. In fact, assuming that all aa occur with the same frequency, the theoretical probability of a sequence of five aa occurring in two proteins can be calculated as 20 ^−5^ (or 1 in 3,200,000 or 0.0000003125), that is, it is extremely low.
- Peptide sharing involves almost all viral proteins and human proteins linked to immunodeficiencies. Exceptions are the viral ORFs 7, 8, and 10 and human proteins CD40L, CD81, ICOS, IL21, RFXAP, and SH21A that were found to be extraneous to the peptide sharing
- Furthermore, the pentapeptide overlap detailed in Table 1 is highly specific for SARS-CoV-2. As reported in Table 2 , none of the 118 shared pentapeptides are present in the pathogenic MERS-CoV, 51 and only a few are found in the mildly pathogenic human coronavirus HCoV-OC43, as well as in HCoV-229E which cause only mild symptoms. 52

At first glance, Table 1 shows that viral matches are disseminated among human proteins that are interconnected in complex pathways, involved in multiple fundamental roles in immune regulation, and linked to defects in activation/development of B and T lymphocytes. An example is CAR11, a protein that plays a key role in the adaptive immune response by transducing NFKB activation downstream of TCR and BCR involvement, so that CAR11 alterations lead to defects in T-, B-, and NK-cell function and to immunodeficiencies. 53 54 Genetic inactivation of the gene *CARD11* results in a complete block in T- and B-cell immunity as CAR11 is essential for antigen receptor- and protein kinase C–mediated proliferation and for cytokine production in T- and B-cells. 55 The regulation of CAR11 signaling is a critical switch governing the decision between death and proliferation in antigen-stimulated mature B-cells. 56 Indeed, CAR11 deficiency causes profound combined immunodeficiencies in human subjects. 57 Nor are all the other proteins involved in the peptide sharing and summarily described in Table 1 of less importance in governing and regulating the immunity status.

However, space constraints do not allow for a one-by-one analysis of all human proteins listed in Table 1 , and only some of the tabulated human proteins will be discussed below.

### CD19, CD20, CD27, and CD70

The cluster differentiation molecules CD19, CD20, CD27, and CD70 are involved in the development, differentiation, activation, and survival of B-cell lymphocytes. 58 In particular, CD19 is not required for B-cell production, but the absence of CD19 inhibits the full activation and maturation of B-cells, thus causing panhypogammaglobulinemia in the presence of a normal number of B-cells in the blood. 59 Also CD20 deficiency can lead to hypogammaglobulinemia in the presence of a normal number of B-cells. 60 Defects in the CD27–CD70 axis indicate an immunodeficiency associated with terminal B-cell development defect and immune dysregulation leading to autoimmunity, uncontrolled viral infection, and lymphomas. 61

### RAG1 and RAG2

The two recombination-activating RAG1 and RAG2 proteins are essential for generating the immune response. Indeed, RAG1 and RAG2 synergistically preside over the genomic rearrangements that initiate the molecular processes that lead to lymphocyte receptor formation through V(D)J recombination. Variants in RAGs are common genetic causes of immunodeficiencies. 62 63 64

### NFKB1 and NFKB2

NFKB1 and NFKB2 share 20 pentapeptides with the SARS-CoV-2 proteome ( Table 1 ). NFKB1 and NFKB2 are examples par excellence of proteins that, if hit by cross-reactions, can cause the decline in the anti-SARS-CoV-2 immune responses. Alterations of NFKB1 are a common cause of immunodeficiency. The clinical phenotype of NFKB1 deficiency includes hypogammaglobulinemia and sinopulmonary infections, as well as other highly variable individual manifestations. 65 In particular, alterations in the expression of the NFKB1 subunit p50 is associated with immunodeficiency. 66

Similarly, NFKB2 is involved peripheral lymphoid organ development, B-cell development and Ab production, 67 and alterations in the p52 subunit appear to be specifically involved in Ab deficiency. Indeed, p52-deficient animals (1) have reduced numbers of B-cells and consistent with a loss of B-cell follicles, (2) are unable to form germinal centers and are impaired in Ab responses to T-dependent antigens, and (3) lack follicular dendritic cell networks. 68 As a matter of fact, coordination between p50 and p52 is essential in the development and organization of secondary lymphoid tissues, 69 that is, the sites where naive lymphocytes mature and initiate an adaptive immune response. 70 Emblematically, a single p52 nucleotide mutation, a nonsense mutation creating a premature stop codon (pos.W270), was found to be associated with haploinsufficiency and Ab deficiency. 71

Therefore, it is relevant that many of the pentapeptides shared by NFKB1 and NFKB2 with the viral proteome (i.e., 10 out of 20) are allocated in the two subunits p50 and p52 ( Supplementary Table S2 .

### Immunological Potential of the Viral versus Human Peptide Sharing: NFKB as an Example

The extensive sharing of minimal immune determinants between the virus and NFKB1/NFKB2 and the associated potential for cross-reactivity might be able to block the physiological functioning of NFKB1 and NFKB2, resulting in the immunosuppression that follows exposure to SARS-CoV-2. 2 3 4 5 6 7 8 9 10 11 12 13 14 15 16 17 18 19 20 21 22 23 A solid support for this possibility is given by the analysis of the immunological potential of the peptide overlap between the SARS-CoV-2 proteome and the two proteins NFKB1 and NFKB2. Indeed, Table 3 documents that, according to IEDB, 50 the 20 pentapeptides that are common to the virus and NFKB1/NFKB2 ( Table 1 ) are also found in numerous SARS-CoV-2-derived epitopes that have been experimentally validated as immunoreactive in the human host.

**Table 3:** Immunoreactive SARS-CoV-2-derived epitopes containing pentapeptides shared between SARS-CoV-2 and NFKB1/NFKB2

IEDB ID a	Epitope sequence b	IEDB ID a	Epitope sequence b
2432	alallLLDRL	1397276	ersgarskqrRPQGL
34851	lallLLDRL	1397409	rskqrRPQGLpnnta
37473	lLLDRLnql	1452222	iksqDLSVVskvvkv
37515	llLLDRLnql	1490109	pSVGPKqaslngvtl
39582	lspvALRQMscaagt	1500188	rskqrRPQGLpnnt
45385	npKTPKYKf	1513800	tfggpsDSTGSn
1074903	gdaalallLLDRLnql	1539491	alallLLDRLnqles
1075018	qELIRQgtdykhw	1539750	crkvqhmvvKAALLa
1149886	ismatnyDLSVVnar	1539768	cvdipgiPKDMTyrr
1310320	daalallLLDRLnql	1539806	ddfveiiksqDLSVV
1310358	eiiksqDLSVVskvv	1539824	deismatnyDLSVVn
1310598	llLLDRLnqleskms	1539833	DFKLNeeiaiilasf
1311682	garskqrRPQGLpn	1539942	dqELIRQgtdykhwp
1312093	aalallLLDRLnqle	1540048	eehfietISLAGsyk
1313309	pritfggpsDSTGSn	1540103	ELIRQgtdykhwpqi
1313389	qtqgnfgdqELIRQg	1540137	eqtqgnfgdqELIRQ
1313478	RPQGLpnntaswfta	1540169	evkilNNLGVdiaan
1313538	sDSTGSnqngersga	1540456	ggdaalallLLDRLn
1313553	sgarskqrRPQGLpn	1540513	glqpSVGPKqaslng
1313575	skqrRPQGLpnntas	1540692	hLLLVAAGleapfly
1313745	tISLAGsyk	1540751	icqavtANVNAllst
1315885	ELIRQgtdy	1540773	ietISLAGsykdwsy
1316419	fgdqELIRQgtdykh	1541014	kilNNLGVdiaantv
1316834	fnicqavtANVNAll	1541102	kpvpevkilNNLGVd
1318946	ISLAGsykdw	1541163	kvninivgDFKLNee
1323201	qELIRQgtdy	1541346	lkvdtanpKTPKYKf
1324011	RPQGLpnnta	1541368	lLLDRLnqleskmsg
1325450	tfggpsDSTGSnqng	1541425	lNNLGVdiaantviw
1332121	gnfgdqELIRQgtdy	1541700	nelspvALRQMscaa
1332637	LLDRLnq	1541742	ninivgDFKLNeeia
1342979	llLLDRLnqle	1541745	nivgDFKLNeeiaii
1377619	alallLLDRLnqlesk	1542039	pvALRQMscaagttq
1377643	allLLDRLnqleskms	1542155	qnnelspvALRQMsc
1377838	arskqrRPQGLpnnt	1542618	svfnicqavtANVNA
1378299	daalallLLDRLnqle	1542868	tpeehfietISLAGs
1381105	ggdaalallLLDRLnq	1543037	vdtanpKTPKYKfv
1381497	gnggdaalallLLDRL	1543087	vgDFKLNeeiaiila
1384139	lallLLDRLnqleskm	1543263	vtANVNAll

Abbreviations: IEDB, Immune Epitope DataBase; NFKB, nuclear factor kappa B; SARS-CoV-2, severe acute respiratory syndrome-coronavirus-2.

a Epitopes listed according to the IEDB ID number. Further details and references for each epitope are available at: *www.iedb.org/* . 50

b Shared peptides are given capitalized.

## Conclusion

Considerable information is presently available on the immune responses evoked by SARS-CoV-2 passive/active infection. Nevertheless, a main question remains unanswered, that is, why higher levels of anti-SARS-CoV-2 immune responses characterize COVID-19 patients who had a severe disease course or died compared with patients who had a mild COVID-19 course and recovered. Here, the data shown in Tables 1 and 3 locate the key to this immunological contradiction in the immune responses themselves which have the pathogenic potential to cross-react with self-proteins profoundly involved in the generation of the humoral and cellular adaptive immunity. Hence, the data have significant scientific implications, as they offer the molecular truth of peptide sharing and the resulting cross-reactivity as a likely mechanistic basis for understanding and explaining how the human anti-SARS-CoV-2 immune responses are overruled. More generally, the data offer a logical explanation for the currently still obscure phenomenon of virus-induced immunosuppression which can effectively be defined as a molecular mimicry syndrome.

Clinically, it derives from the above that the severity of the COVID-19 course is related to the extent of the anti-SARS-CoV-2 primary and secondary immune responses. Indeed, the more massive and avid is the immune response triggered by the virus, the more massive and intense can be the self-attacks against the human proteins that generate, modulate, and preside over the defensive adaptive immune response, And obviously, conversely, the less intense are the immune responses, and less intense are the cross-reactivity and the immunosuppression with consequent positive outcomes of SARS-CoV-2 disease.

As conclusive notes, the present study (1) warrants a global effort to thoroughly testing COVID-19 patients' sera for auto-Abs against the broad molecular peptide platform outlined in Table 1 , and (2) implies that immunotherapeutic strategies based on repeated boosters might unlikely be appropriate and successful in the current pandemic, and indeed might aggravate the immunosuppression pathology.

Finally and of utmost importance, this study once again indicates that using entire pathogen antigens in immunotherapies can associate with cross-reactivity and lead to autoimmune manifestations. The use of the peptide uniqueness concept remains the main scientific path for designing safe and effective therapeutic approaches against infectious agents. 44 45 72
