# Supplementary material for: SARS-CoV-2-Induced Immunosuppression: A Molecular Mimicry Syndrome
Source: Glob Med Genet. 2022 Jul 14;9(3):191–9. doi: 10.1055/s-0042-1748170 (PMC9282940; doi:10.1055/s-0042-1748170)
Supplement: Supplementary file 1 — Supplementary Material [file 10-1055-s-0042-1748170-s2200008.pdf]

Supplementary Table S1 List of 38 human proteins retrieved from UniProt database using the keywords "immunodeficiency hypogammaglobulinemia AND reviewed"

ALG12; BCL10; BTK; C2TA; CAR11; CD19; CD20; CD27; CD40L; CD70; CD81; CR2; CTLA4; CXCR4; I2BP2; ICOS; IKZF1; IL21; IRF9; KPCD; LAT; MOES; NFKB1; NFKB2; NS1BP; P85A; RAG1; RAG2; RFX5; RFXAP; RFXK; SH21A; SP110; TR13B; TR13C; TRNT1; VAS1; XIAP

Note: Proteins given by UniProt entry.

 © 2022. The Author(s).

Supplementary Table S2 Pentapeptide sharing between the SARS-CoV-2 proteome and the NFKB p50 and p52 subunits[a]

p50, aa 1-433
maeddpylgrpeqmfhldpslthtifnpevfqpqmalptdgpylqileqpkqrgfrfryvcegpshgglpgasseknkksypqvkicnyvgpakvivqlvtngknihlhahslvgkhcedgictvtagpkdmvvgfanlgilhvtkkkvfetlearmtea<br>cirgynpgllvhpdlaylqaegggdrqlgdrek**ELIRQ**aalqqtkem**DLSVV**rlmftaflp**DSTGS**ftrrlepvvsdaiydskapnasnlkivrmdrtagcvtggeeiyllcdkvqkddiqirfyeeeenggvwegfgdfsptdvhrqfaivf**KTPKYK**<br>dinitkpasvfvqlrrksdletsepkpflyypeikdkeevqrkrqklmpnfsdsfgggsgagaggggmfgsggggggtgstgpgysfphygfptyggitfhpgttksnagmkhg
p52, aa 1-454
mescynpgldgiieyd**DFKLN**ssivepkepapetadgpylviveqpkqrgfrfrygcegpshgglpgassekgrktyptvkicnyegpakievdlvthsdpprahahslvgkqcselgicav**SVGPKDMT**aqf**NNLGV**lhvtkknmmgtmiqklq<br>rqrlrs**RPQGL**teaeqreleqeakelkkvmdlsivrlrfsaflrasdgsfslplkpvisqpihdskspgasnlkisrmdktagsvrggdevyllcdkvqkddievrfyeddengwqafgdfsptdvhkqyaivfrtppyhkmkierpvtvflqlkrkrggdvsdsk<br>qftyyplvedkeevqrkrrkalptfsqpfgggshmgggsggaaggyggaggggslgffpsslayspyqsgagpmgcypgggggaqmaatvpsrdsgeeaaepsapsrtpqcepqapemlqrareynarlfgla

Abbreviations: NFKB, nuclear factor kappa B; SARS-CoV-2, severe acute respiratory syndrome-coronavirus-2.
[a]Shared peptides given capitalized and bold.

 © 2022. The Author(s).
